# Individual, family and social-related factors of eating behavior among Chinese children with overweight or obesity from the perspective of family system

**DOI:** 10.3389/fped.2024.1305770

**Published:** 2024-02-22

**Authors:** Hanfei Zhu, Kang Zhao, Lidong Huang, Wenbing Shi, Chulei Tang, Ting Xu, Shuqin Zhu, Qin Xu, Xiaonan Li, Yinhua Chen, Qianqi Liu, Linhui Yang

**Affiliations:** ^1^ School of Nursing, Nanjing Medical University, Nanjing, China; ^2^ Department of Child Health Care, Children's Hospital of Nanjing Medical University, Nanjing, China

**Keywords:** pediatric obesity, eating behavior, influencing factors, family system theory, cross-sectional study

## Abstract

**Purpose:**

The purpose of the present study is to examine the factors contributing to the development of eating behavior in overweight and obese children from the perspective of the family system.

**Methods:**

A cross-sectional survey was conducted by using convenience sampling method to select 388 participants in two primary schools in Jiangsu, China. Individual, family and social-related factors were collected. Individual factors included age, gender, ethnicity, single child, social anxiety, depression, physical activity, sleep duration, screen time. Family factors included family environment, family structure, family function, family income, parenting style, parental feeding behavior, home food environment and marital satisfaction. Social-related factors included place of residence, number of surrounding restaurants and social support. Univariate analysis, correlation analysis and multivariate analysis were used to identify factors of eating behavior among Chinese children with overweight and obese.

**Results:**

In this study, 388 participants took part with a 94.865% response rate. In the univariate analysis, the significant differences regarding Dutch Eating Behavior Questionnaire (DEBQ) scores were found between children aged 6–9 years and those aged >9 years. Correlation analysis indicated that parent's nutrition literacy (*r* = 0.118, *P* < 0.05), pressure to eat (*r* = 0.212, *P* < 0.01), perception of child weight (*r* = −0.112, *P* < 0.05) and family function (*r* = −0.563, *P* < 0.01) were associated with children's eating behavior. With regard to psychosocial factors, children's social anxiety (*r* = 0.299, *P* < 0.01) and depressive symptoms (*r* = 0.081, *P* < 0.05) were in positive correlation with eating behavior. The independent variables included in the initial model were age, father's employment status, social anxiety, maternal punishment and harshness, parents' nutrition literacy, pressure to eat, family function and perception of child weight. These variables in the final model accounted for 20.7% of the variance.

**Conclusion:**

We found that age, father's employment status, social anxiety, maternal punishment and harshness, parents' nutrition literacy, pressure to eat, family function and perception of child weight have great effect on children's eating behavior who are overweight or obese. As early childhood is a critical timeline for child development, children's social anxiety, parenting style, parent's nutrition literacy, parent's feeding behavior and family function should be intervened to promote eating behavior. Intervention programs aimed at promoting healthy eating behaviors among children, thereby mitigating the risk of pediatric obesity, should primarily target parents.

## 1 Introduction

Obesity has become a global public health problem, and the World Health Organization (WHO) has identified it as one of the most serious diseases threatening human health in the 21st century (1). Childhood obesity has gradually become a growing concern in all over the world in recent years. According to the Report on Nutrition and Chronic Diseases of Chinese Residents (2020) (2), the overweight and obesity rates of children and adolescents under the age of 6 and 6–17 in China have reached 10.4% and 19.0%, respectively. Without taking intervention measures, the prevalence of overweight and obesity in children would reach 28%, with a total of 49.48 million children affected by 2030 (3). Childhood obesity can lead to impairments in bodily organ function, diminished mobility, and decreased physical fitness. Moreover, it has the potential to induce psychological and behavioral problems, such as social adjustment disorders and behavioral disorders, consequently leading to detrimental impacts on the holistic physical and mental health of children (4). Approximately half of the children who experience obesity in their early years are likely to persist in their obese state throughout adulthood (5), which will significantly increase the likelihood of developing diabetes, cardiovascular disease, cancer, reproductive disorders, and various other ailments (6). The rates of obesity have been rapidly rising in children and adolescents, along with the growing burden of disease and disability, will induce social and economic consequences for society, not only raising health-care expenditures but also impeding economic growth (7). A series of policies have been introduced at the national level to actively prevent and regulate children's nutrition issues in China. The “Healthy China 2030” Outline (8) explicitly recommends strengthening the control of children's diet and nutrition as well as promoting a healthy weight plan. The Implementation Plan for Prevention and Control of Childhood and Adolescent Obesity, which was jointly released in 2020 by the General Office of the National Health and Health Commission, the General Office of the Ministry of Education, and six other departments, calls for close collaboration between families, schools, and medical institutions, as well as for vigorously populating nutrition and health knowledge and optimizing weight management services for children and adolescents (9). Thus, it is clear that the issue of children overweight and obesity is serious and urgent, indicating that there is a pressing need for scientific promotion of pediatric obesity prevention and control.

Childhood plays a pivotal role in an individual's overall growth and development. The nutritional status experienced during this period not only impacts children's growth and development, but also has long-lasting effects on their health in adulthood (10). Extensive research has demonstrated that childhood obesity is influenced by a complex interplay of genetic, environmental, and other factors (11). Among these factors, eating behavior emerges as a particularly significant intervention point for the occurrence and progression of obesity. Children's various activities and psychological development cannot be separated from the interaction with the environment and the occurrence of children's behavior problems is the result of individual, family and social factors (12). Of these factors, the family unit serves as the initial milieu for a child's social development, wherein they acquire early life experiences and establish their initial behavioral patterns, attitudes, and beliefs (13). Given that children typically possess a limited social relationship, the family assumes a crucial role in the development of children's prosociety and their behavior.

The influence of the several intrinsic components has a synergistic effect on family as well. Previous studies found that the development of eating behavior in children was directly related to family structure, family economic status, family environment, family function, parent-child system, and the marital system (14, 15). Compared to fathers, mothers were the primary caregivers in the household (16). Additionally, the fact that grandparents live with their grandchildren is a common family structure in China, which increases the phenomena of grandparents gradually participating in family feeding, while it also links to childhood obesity (17). Home food environment influences children's eating behavior and nutritional status (18). Parents' comprehension of nutrition knowledge, level of education, and financial situation are related to children's food preferences (19). The findings of a cross-sectional study suggest that children raised by grandparents exhibit more nutritional imbalance and quicker eating speed (20). Besides, grandparents usually force children to eat and eliminate food waste, which leads to higher rates of obesity (20). These results highlight the significant influence parents have on their children's eating habits through their own dietary patterns, parenting style, attitudes towards parenting, and food preferences. Consequently, it is crucial for parents to actively participate in the establishment and regulation of their children's eating behavior. Existing studies have examined the influencing factors on various dimensions of eating behavior. However, there is a dearth of comprehensive scientific understanding specifically pertaining to obese children, as most studies have only explored factors from a singular dimension. Therefore, it is imperative to address the urgent issue of comprehensively analyzing the factors influencing obese children's eating behaviors, considering individual, family and social-related factors.

Some researchers currently consider that the theory and practice of weight control for children based on the family system is valuable, each family member impacts and is also affected by other family members since families are interconnected systems (21). Based on the principles of family system theory, the whole is greater than the sum of its parts. This implies that the dynamics encompassing the entire family unit, including the interactions between couples, parents, and children, bear substantial implications for the improvement of familial relationships and the facilitation of behavioral modifications (22). As a key member of the family system, children's behavior characteristics and inner attitudes and beliefs are greatly influenced by other family members. The interplay among family members collectively shapes the overall functioning of the family system, commonly referred to as family function, and any alterations in the composition of family members inevitably lead to consequential changes in the family as a whole. Kathrin et al. found a positive relationship between family cohesion and external eating behavior of children and a negative relationship between family cohesion and restrained eating of children (23). Cui Ying et al. showed that the degree of expressiveness, intellectual-cultural orientation and active-recreational were lower than control group,and the degree of family conflict and control were higher in children with obesity (24). These studies revealed that the whole family structure, family environment or family function may be related to children's eating behavior. The eating behavior of children may be influenced from a range of internal and external factors, thereby rendering family system theory a viable framework for its explication. However, the specific factors that significantly influence eating behavior remain unknown. To gain a comprehensive and systematic understanding of the predisposing factors in children with overweight or obesity, a cross-sectional survey was conducted to elucidate their role and effect. The objective of the present study is to examine the factors contributing to the development of eating behavior in overweight and obese children from the perspective of the family system.

## 2 Materials and methods

### 2.1 Participants

The present study was of a cross-sectional design. Our sample consisted of primary school students in Nanjing. A convenience sampling method was used. Samples were included due to the inclusion criteria: (1) aged 6 to <12 years; (2) used Chinese criteria defined by the National Health Commission of the People's Republic of China to screen overweight and obesity. Detailed criteria is attached in the Supplementary Materials. Exclusion criteria were: (1) patients with a systemic or metabolic disease, such as hyperthyroidism, hypothyroidism, diabetes mellitus type I, and Cushing's syndrome; (2) patients with a diagnosis of intellectual disability, attention deficit hyperactivity disorder (ADHD), developmental disability, nondevelopmental speech or language deficits; (3) using medication drugs, such as glucocorticoids, dexmethylphenidate hydrochloride, dextroamphetamine sulfate, lisdexamfetamine dimesylate, methamphetamine hydrochloride, methylphenidate, and methylphenidate hydrochloride.

### 2.2 Procedures

The purpose and significance of the investigation was explained ahead to the principals and teachers. Questionnaires were distributed online to parents through Electronic “Wen Juan Xing” tool (https://www.wjx.cn/). Individual, family and social-related factors measured in this study are listed in Table 1. The parents were informed about the aim of the study and a parental written consent was obtained for each child using a unified instruction. All the questionnaires were finished by the parents and their children on a voluntary basis. The questionnaire was a self-administered type of questionnaire, divided into two sections, each for parents and each for children. Height and body weight were self-reported by parents, and the body mass index (BMI) was calculated. The questionnaires were checked for completeness and any incomplete questionnaire would be discarded. All samples were collected according to protocols approved by the Committee of Nanjing Medical University (Ethical approval number: 2021-538). All participants included in the study provided written informed consent before investigation.

**Table 1:** Individual, family and social-related factors measured in the study.

	Factors
Individual factors	Age, Gender, Ethnicity, Single child, Puberty status
	Social anxiety (27), Depression (29)
	Physical activity, Sleep duration, Screen time
Family factors	Family environment (31), Family structure, Family function (32), Family income
	Parenting style (33), Parental feeding behavior (34), Home food environment (18)
	Marital satisfaction (35)
Social-related factors	Place of residence, Number of Surrounding restaurants, Social support (36)

### 2.3 Measures

#### 2.3.1 General infromation and socio-demographic questionnaire

The researchers designed the general information and socio-demographic questionnaire. The quesetionnaire was reported by parents. The general information includes children's age, gender and ethnicity. The socio-demographic characteristics include family structure, parents' educational level, parents' work status and place of residence. Besides, the information about the puberty status was extracted from the medical record and it was determined by a pediatrician experienced in the assessment of secondary sex characteristics according to the method of Tanner.

#### 2.3.2 Dutch eating behavior questionnaire

Eating behavior was assessed using a validated Chinese version of the Dutch Eating Behavior Questionnaire (DEBQ-C), which was translated by Zhao et al. (25). This tool was used to assess three components of eating behavior: emotional, external, and restrained eating (26). All items were rated on a 3-point scale ranging from 1 (never) to 3 (often). The Cronbach's α for this scale was 0.78 and split-half reliability was 0.68. Higher scores suggest a higher level of emotional, external, and restrained eating.

#### 2.3.3 Social anxiety scale for children

Regarding the Social Anxiety Scale for Children (SASC) (27), we employed the Chinese version of the SASC to evaluate children' social anxiety (28). The scale consisted of 10 items and 2 dimensions. SASC was found to have good psychometric properties, with adequate reliability and validity in Chinese population.

#### 2.3.4 Depression self-rating scale for children

Depressive symptoms were assessed using the Depression Self-rating Scale for Children (DSRSC) (29). Answers are given on a three-grade scale (0 = never, 1 = sometimes, 2 = often). The Chinese version of the DSRSC has shown good psychometric properties.

#### 2.3.5 International physical activity questionnaire

Physical activity was measured with the short Chinese version of the International Physical Activity Questionnaire (IPAQ-C) (30). It has been widely used in children by assessing the time spent walking, sitting, performing moderate-intensity physical activity, and performing vigorous-intensity physical activity.

#### 2.3.6 Family environment scale

The Chinese Version of Family Environment Scale has 27 items and 3 subscales, which are family cohesion, family expressiveness and family conflict. The internal consistency reliability for the total scale was 0.937. The split half reliability is 0.505, and the retest reliability is 0.532, which showed good validity and reliability in Chinese students (31). A higher score represents more positive family impact.

#### 2.3.7 Home food environment scale

Home Food Environment Scale was developed by Xiao Su et al, which consisted of 5 subscales (home feeding pattern, home food rules, family eating behavior, parent's nutrition literacy, home food availability) (18). The reliability and validity of the tool are adequate and it may be a useful tool in measuring the home food environment of school-aged children.

#### 2.3.8 Family APGAR index

Family function was measured by the Chinese version of the Family APGAR Index (adaptability, partnership, growth, affection, resolve) (32). Each item was given a grade using a 3-point grading scale. The answer of “often” would receive 2 points, “sometimes” with 1 point and “never” with 0 points. A higher score indicates better family function. In this study, the Cronbach's α for the scale was 0.819.

#### 2.3.9 One's memories of upbringing

Parenting Styles were measured by the Chinese version of the Short-Form of the Egna minnen av Barndoms uppfostran (One's Memories of Upbringing) (s-EMBU-c), which was adapted by Xin Song and was tested good reliability and validity (33). This scale consists of two parts of paternal parenting and maternal parenting (emotional warmth and understanding, excessive interference, punishment and harshness, rejection and denial). All the items were rated on a 4-point scale: never = 1, seldom = 2, occasionally = 3, and often = 4. Higher scores indicate parents' more tendency to adopt the parenting style.

#### 2.3.10 Child feeding questionnaire

Parental control over child feeding as well as parental perceptions and concerns about obesity related to child feeding can be measured using the Child Feeding Questionnaire (CFQ), which was developed by Birch et al. (34). The Chinese version of CFQ is consisted of 8 dimensions and 32 items.

#### 2.3.11 Kansas marital satisfaction scale

A three-item Kansas Marital Satisfaction Scale (KMSS), which can be completed on a seven-point Likert scale ranging from 1 (extremely dissatisfied) to 7 (extremely satisfied), was used to assess marital satisfaction (35).

#### 2.3.12 Social support rating scale

The Social Support Rating Scale (SSRS) was compiled in 1986 by Chinese scholar Xiao Shuiyuan and it is consisted of 10 items (36). SSRS could be applied to assess subjective support, objective support and support utilisation.

### 2.4 Data analysis

The statistical analysis was conducted using SPSS Statistics, version 25.0 (IBM Corp), with *P* ≤ 0.05 considered significant. Descriptive statistics were calculated and expressed as means (standard deviations) for continuous variables, and frequencies (percentage) for categorical variables. The distribution was verified for its normality using the Shapiro–Wilk test. Differences between two groups were analyzed using a *t*-test or one-way ANOVA test for continuous data and the *χ*^2^ or Fischer's exact test for categorical data. Pearson's correlation analysis was used to analyze the correlation among continuous variables and eating behavior. Multiple linear regression analyses with forward stepwise selection were performed to analyze the effects of factors on eating behavior. In multiple linear regression analysis, potential variables which were associated with eating behavior (*P* < 0.1) in univariate analysis were entered, then the independent variables were entered. The indicators provided in the regression models included *R*^2^, adjusted *R*^2^, *R*^2^ change, *F*-value and standardization regression coefficient (β).

## 3 Results

### 3.1 Characteristics of study population

In this study, a total of 409 questionnaires were distributed, and 388 questionnaires were effectively recovered, with an effective recovery rate of 94.865%. Overall, 388 students (228 boys, 58.763%) with an average age of 9.224 ± 1.612 participated in the study. The majority of participants were living with parents (53.351%) and in more than half of the families both parents had a job. Most of the families resided in urban areas (84.728%). Characteristics of the sample and descriptive statistics of the key variables addressed in the study are presented in Tables 2, 3.

**Table 2:** Characteristics of children participated in the survey.

Item	Category	No. (%)/Mean ± SD
Gender		
	Male	228 (58.763)
	Female	160 (41.237)
Age (years)		9.224 ± 1.612
Height (cm)		140.553 ± 12.304
Weight (kg)		44.023 ± 10.542
BMI (kg/m^2^)		22.042 ± 3.069
Ethnicity		
	Han ethnicity	372 (95.876)
	non-Han ethnicity	16 (4.124)
Single child		
	Yes	251 (64.691)
	No	137 (35.309)
Puberty stage		
	Stage I	293 (75.515)
	Stage II	58 (14.949)
	Stage III	32 (8.247)
	Stage IV	5 (1.289)
	Stage V	0

**Table 3:** Characteristics of the families participated in the survey.

Item	Category	No. (%)
Father's educational level		
	Middle school or below	35 (9.021)
	High school	90 (23.196)
	College or Bachelor's degree	246 (63.402)
	Master's degree or above	17 (4.381)
Mother's educational level		
	Middle school or below	32 (8.247)
	High school	100 (25.773)
	College or Bachelor's degree	249 (64.175)
	Master's degree or above	7 (1.805)
Father's employment status		
	Employed	379 (97.680)
	Unemployed	9 (2.320)
Mother's employment status		
	Employed	329 (84.794)
	Unemployed	59 (15.206)
Marital status		
	Married	383 (98.711)
	Divorce or widowhood	5 (1.289)
Monthly income of family (yuan)		
	3,000 or below	11 (2.835)
	3,000–6,000	35 (9.021)
	6,000–8,000	44 (11.340)
	8,000–10,000	66 (17.010)
	10,000 or above	232 (59.794)
Family structure	Live with grandparents and parents	168 (43.299)
	Live alone with parents	207 (53.351)
	Other situations	13 (3.350)
Place of residence		
	Countryside	61 (15.722)
	City or town	327 (84.278)

### 3.2 Influence of general demographic factors, social-demographic factors, behavioral factors and eating behavior

There were no differences in the DEBQ scores according to general demographic factors, social-demographic factors and behavioral factors except age. In the univariate analysis, the significant differences regarding DEBQ scores were found between children aged 6–9 years and those aged >9 years. The results of univariate analysis of general demographic factors, social-demographic factors, behavioral factors and eating behavior are detailed in Table 4.

**Table 4:** Univariate analysis of general demographic factors, social-demographic factors, behavioral factors and DEBQ scores.

Item	Category	DEBQ Score ($\bar{x}\pm s$)	*t*/*F*-Value	*P*-Value
Gender				
	Male	35.965 ± 6.750	−0.067	0.947
	Female	36.012 ± 7.055		
Age (years)				
	6–<9	34.654 ± 6.147	−3.702	<0.001
	9–12	37.197 ± 7.271		
Ethnicity				
	Han ethnicity	35.967 ± 6.870	−0.232	0.823
	non-Han ethnicity	36.375 ± 7.032		
Single child				
	Yes	35.816 ± 6.867	−0.651	0.515
	No	36.286 ± 6.872		
Number of restaurants near home	Few	35.438 ± 6.496	0.710	0.492
	Moderate	35.513 ± 7.143		
	Many	36.344 ± 6.812		
Place of residence				
	Countryside	36.246 ± 7.004	1.752	0.081
	City or town	34.568 ± 5.942		
Physical activity	Low	36.276 ± 6.762	0.510	0.601
	Moderate	36.217 ± 6.620		
	High	35.502 ± 7.264		
Sleep duration (hour)	>10	35.982 ± 6.606	−0.021	0.983
	≤10	35.986 ± 7.062		
Screen time (hour)	≤2	35.867 ± 6.522	1.012	0.312
	>2	37.032 ± 9.386		
Puberty stage				
	Stage I	35.414 ± 6.000	1.298	0.275
	Stage II	35.103 ± 6.106		
	Stage III	33.625 ± 5.154		
	Stage IV	38.200 ± 3.962		

DEBQ: Dutch Eating Behavior Questionnaire.

### 3.3 Influence of family factors and eating behavior

There were no differences in the DEBQ scores between groups categorized by basic family-related factors, which were included parents' educational level, parents' employment status, marital status, monthly income of family, family structure and children's primary caregiver. Table 5 shows the results of the univariate analysis of family factors.

**Table 5:** Univariate analysis of family factors and DEBQ scores.

Item	Category	DEBQ Score ($\bar{x}\pm s$)	*t*/*F*-Value	*P*-Value
Father's educational level			2.478	0.061
	Middle school or below	35.687 ± 7.754		
	High school	36.033 ± 6.423		
	College or Bachelor's degree	35.712 ± 6.676		
	Master's degree or above	40.354 ± 8.938		
Mother's educational level			1.883	0.132
	Middle school or below	36.002 ± 7.718		
	High school	35.151 ± 5.796		
	College or Bachelor's degree	36.172 ± 7.023		
	Master's degree or above	41.141 ± 9.769		
Father's employment status			1.915	0.056
	Employed	36.085 ± 6.834		
	Unemployed	31.665 ± 7.668		
Mother's employment status			0.105	0.917
	Employed	36.002 ± 6.987		
	Unemployed	35.903 ± 6.214		
Marital status			−0.224	0.829
	Married	36.024 ± 5.813		
	Divorce or widowhood	36.502 ± 5.879		
Monthly income of family (yuan)			0.492	0.742
	3,000 or below	33.272 ± 7.404		
	3,000–6,000	35.624 ± 6.172		
	6,000–8,000	36.086 ± 6.876		
	8,000–10,000	36.292 ± 7.186		
	10,000 or above	36.064 ± 6.882		
Family structure	Live with grandparents and parents	36.062 ± 6.880	0.437	0.646
	Live alone with parents	35.584 ± 7.063		
	Other situations	33.402 ± 4.934		
Children's primary caregiver	Mother	35.934 ± 6.982	0.577	0.631
	Father	36.922 ± 6.210		
	Grandparents	35.584 ± 7.064		
	Others	33.402 ± 4.932		

### 3.4 Relationships between eating behavior and continuous variables

Table 6 displays the correlation analysis results for continuous variables. Correlation analysis indicated that parent's nutrition literacy (*r* = 0.118, *P* < 0.05), pressure to eat (*r* = 0.212, *P* < 0.01), perception of child weight (*r* = −0.112, *P* < 0.05) and family function (*r* = −0.563, *P* < 0.01) were significantly associated with children's eating behavior. With regard to psychosocial factors, children's social anxiety (*r* = 0.299, *P* < 0.01) and depressive symptoms (*r* = 0.081, *P* < 0.05) were in positive correlation with eating behavior. Relationship between parenting styles and eating behavior was also shown a positive tendency. Mothers' excessive interference (*r* = 0.141, *P* < 0.01) and rejection and denial (*r* = 0.178, *P* < 0.01) were significantly associated with children's eating behavior. Besides, significant associations were found between maternal punishment and harshness and eating behavior (*r* = 0.148, *P* < 0.01). Fathers' excessive interference (*r* = 0.141, *P* < 0.01) and rejection and denial (*r* = 0.149, *P* < 0.01) were significantly associated with children's eating behavior. The results of these analyses are summarized in Table 6.

**Table 6:** Correlation analysis of children's eating behavior and continuous variables.

	Pearson coefficient			
	Eating behavior	Emotional eating	Restrained eating	External eating
Home food availability	0.039	0.042	−0.051	0.083
Home food rules	0.063	0.064	0.033	0.041
Family eating behavior	0.0001	0.015	0.026	−0.041
Parent's nutrition literacy	0.118*	0.112*	0.099	0.058
Restriction	0.063	0.021	0.041	0.117*
Monitoring	−0.007	−0.036	0.006	0.022
Pressure to eat	0.212**	0.193**	0.124*	0.237**
Perceived responsibility for feeding	0.082	0.017	0.135**	0.062
Perception of parent weight	−0.092	−0.010	−0.140**	−0.092
Perception of child weight	−0.112*	−0.058	−0.073	−0.134**
Concern about child weight	0.017	0.004	0.008	0.030
Marital satisfaction	−0.008	−0.030	−0.012	0.029
Family function	−0.563**	−0.394**	−0.338**	−0.378**
Family cohesion	−0.099	−0.075	−0.055	−0.135*
Family expressiveness	−0.091	−0.059	−0.071	−0.116
Family conflict	−0.053	0.012	−0.086	−0.090
Social anxiety	0.299**	0.285**	0.143**	0.233**
Depressive symptoms	0.081*	0.142**	−0.020	0.029
Social support	0.015	0.022	0.075	−0.054
Maternal emotional warmth and understanding	0.004	−0.173**	0.147**	0.108*
Maternal excessive interference	0.155**	0.121*	0.118*	0.119*
Maternal punishment and harshness	0.148**	0.072	0.103*	0.177**
Maternal rejection and denial	0.178**	0.214**	0.04	0.121*
Paternal emotional warmth and understanding	0.065	0.034	0.061	0.061
Paternal excessive interference	0.141**	0.210**	0.101*	−0.016
Paternal punishment and harshness	0.040	0.059	0.011	0.012
Paternal rejection and denial	0.149**	0.200**	0.099	0.020

* Correlation is significant at the 0.05 level, *P *< 0.05.

** Correlation is significant at the 0.01 level, *P *< 0.01.

#### 3.4.1 Multiple linear regression analyses

The multiple linear regression analyses were conducted to examine the relative independent contribution of the variables are presented in Table 7. All the independent variables that were associated with children's eating behavior in univariate analysis (*P* < 0.1) were entered into the multiple regression model. The independent variables included in the initial model were age, father's employment status, social anxiety, maternal punishment and harshness, parents' nutrition literacy, pressure to eat, family function and perception of child weight. These variables in the final model accounted for 20.7% of the variance.

**Table 7:** Multivariate linear regression modeling for factors of eating behavior.

Variable	Partial regression coefficent	Standar error	Standardized regression coefficient	*t*	Significance
Constant	30.075	6.498		4.629	<0.001
Age	1.950	0.644	0.142	3.028	0.003
Father's employment status	−4.505	2.127	−0.099	−2.119	0.035
Social anxiety	0.465	0.079	0.278	5.896	<0.001
Maternal punishment and harshness	0.204	0.077	0.125	2.643	0.009
Parent's nutrition literacy	0.775	0.196	0.204	3.960	<0.001
Pressure to eat	0.257	0.098	0.133	2.629	0.009
Family function	−0.271	0.113	−0.112	−2.390	0.017
Perception of child weight	−0.348	0.151	−0.108	−2.304	0.022

*R*^2 ^= 0.235; adjusted *R*^2 ^= 0.207; *F *= 10.068; *P *< 0.0001.

## 4 Discussion

This study systematically explores influencing factors of eating behavior in Chinese overweight and obese children. We found that age, father's employment status, social anxiety, maternal punishment and harshness, parent's nutrition literacy, parental perception of child weight, pressure to eat and family function, significantly impact the eating behavior of overweight or obese children.

In our study, age emerged as a crucial determinant in shaping children's eating habits, as these behaviors tend to develop early in life and persist into adulthood. However, as children get older, their needs for complementary foods increase and there is an increasing autonomy in the dietary intakes of children (37). This may lead to an increased tendency to buy energy-dense foods. Thus, it is imperative to prioritize the evaluation of eating behavior among primary school students aged 9–12. Some studies have indicated that movement behaviors, which encompass physical activity, sedentary behavior (including screen time), and sleep, play a significant role in the development of childhood overweight and obesity. Consequently, it is crucial to address issues such as physical inactivity, excessive sedentary time, and inadequate sleep as contributing factors to combat this prevalent health concern (38). However, we didn't find that these movement behaviors were associated with children's eating behavior. This may because children are in a period of increasing energy demand, and eating behavior can promote the body's energy intake. Although physical activity is considered as the main factor of energy consumption, it always at a low level in obese children (39).

Besides, the findings of this study indicate that social anxiety significantly contributes to the formation of eating behavior among children affected by obesity. Social anxiety is characterized as the apprehension or distress experienced when subjected to evaluation or scrutiny in social or performance-related situations (40). Children exhibiting social anxiety frequently exhibit heightened levels of nervousness, tension, or panic, particularly in response to interpersonal stressors, and may also harbor concerns regarding the adverse consequences of previous unfavorable encounters and potential negative outcomes in the future. Consistent with the prior research, obese children often experience negative social experiences than their non-overweight counterparts. Thus, social anxiety may be a particularly salient risk factor for body weight concerns and disordered eating (41).

Moreover, no significant differences were noted with respect to family structure in this study. In another word, children who live with grandparents and parents don't have worse eating behavior than children who live alone with parents or in other situations. This is different from previous studies that found children raised by grandparents have higher rates of overweight or obesity (42). The phenomenon that grandparents often participate in family feeding, taking the responsibility for food buying, cooking, feeding, and often participate in the care of grandchildren is common in China (42). However, the data in our study didn't show the effect that family structure is associated with children's eating behavior.

The presence of clear role division, open communication, and good norms within well-functioning families has the potential to foster healthy eating behavior in children. Annette's research further reveals a correlation between family dysfunction, negative family food-related experiences, and an increased likelihood of disordered eating (43). Previous studies have consistently suggested that a positive family system plays a crucial role in establishing and promoting beneficial health behavior, primarily through the mechanisms of role modeling, provision of healthy foods, and support for engaging in healthy eating practices (44).

In the linear regression model, we found the punitive and strict parenting style adopted by mothers is more likely to lead to poor eating behavior in obese children. Prior research has demonstrated that mothers frequently assume the role of feeders when engaging in the process of nourishing their children. Nevertheless, disciplinary and stringent approaches are more inclined to diminish children's resistance towards parental feeding practices. Additionally, certain studies corroborate the notion that authoritative parenting exhibits a positive correlation with improved weight-related outcomes, in contrast to alternative parenting styles (45, 46). A Chinese researcher also found that the scores of parents' emotional warmth and understanding in the obese group were lower than those in the normal group, while the scores of parents' rejection and denial, and excessive interference were significantly higher than those in the normal group (24). This suggests that parental rejection and denial, and excessive interference may be associated with children's weight, which is consistent with our findings. Nicole's et al. cross-sectional findings suggest that authoritative parenting may lead adolescents to adopt healthier eating habits and may be of higher quality, suggesting that parental parenting needs to be considered to enhance the effectiveness of family-based interventions (47). From these studies, we could clarify the relationships between parenting style and eating behavior.

An important finding is that parent's nutrition literacy plays a key role in the development of eating behavior. This reveals that parents who have lower nutrition literacy, their children are more likely to have unhealthy eating behavior, especially emotional eating. High nutrition literacy means that parents are more willing to encourage children to eat fruits and vegetables, focus on nutrition-related information, discuss nutrition-related information, read nutrition labels and have more nutrition knowledge, which may promote children to develop healthy eating behavior (18). Another significant factor in this study is parent's perception of a child's weight. When parents perceived that their child may be overweight or obese, they may adopt some measures to change child's eating behavior. In addition, Lu Xiaoyu et al. found that maternal parenting style affects children's BMI through the perception of children's weight (48). This relationship could also be explored in the future study.

The present results suggest that the social-related factors have limited influence on children's eating behavior. On the one hand, built environment factors affect children's energy expenditure more through recreational activities and commuting mode, so as to regulate their body weight (49). On the other hand, the predominant impact of parental feeding behavior on children's food selection may attenuate the association between the distribution of restaurants and convenience stores in the built environment and children's eating behavior (50). In terms of social support, greater emphasis was placed on extrinsic familial social connections, such as peers and neighbors, and the results indicated a tenuous correlation between social support and children's eating behavior, further underscoring the primary influence of parents on their children's eating habits.

### 4.1 Strengths and limitation

The current study conducted a comprehensive investigation into the impact of individual, family, and social-related factors on the emergence of eating behavior in overweight and obese children in China. The findings offer insights into the conflicting outcomes reported in prior research. By adopting a family system perspective, this study expands upon the individual-centric approach by examining multiple drivers, including individual factors, family systems, and social system factors. There are some new findings that are worth noting. We found that social anxiety is an important influencing factor of pediatric obesity. It is suggested that intervention programs can be designed to reduce children's social anxiety in the future. However, there are some limitations still need to be considered. Firstly, the study primarily utilized convenience sampling from two primary schools in Nanjing, which may lead to bias in the conclusion, weaken the external validity of the results and restrict the generalizability of the research findings. To enhance the scope of future investigations, it is recommended to conduct multi-center surveys across diverse regions. Additionally, due to the characteristics of variables, self-reported measures such as height, weight, physical activity, sleep time, and screen time were predominantly employed. This approach may introduce potential measurement bias in assessing outcomes and affect the correlation coefficient of the variables. Lastly, the utilization of a cross-sectional design may limit the ability to establish strong associations and elucidate the relationships between factors and eating behavior. In addition, since the development of obesity is multi-factorial, some genetic, and social, cultural and economic factors have not been included in the analysis, which is worthy of further exploration in future research. Despite not being a clinical trial, the findings of this study hold significance for the evaluation and mitigation of unhealthy eating behavior in overweight and obese children. Compared with biological variables and macro-level variables (e.g., economic, political, and cultural variables), relative variables of individual and family system are easier to be observed and interfered, which is also the advantage of this study.

## 5 Conclusion

In conclusion, it can be inferred that the determinants of children's eating behavior are likely to be multifaceted and intricate. We found age, father's employment status, social anxiety, maternal punishment and harshness, parents' nutrition literacy, pressure to eat, family function and perception of child weight have great effect on children's eating behavior who are overweight or obese. As early childhood is a critical timeline for child development, children's social anxiety, parenting style, parent's nutrition literacy, parent's feeding behavior and family function should be intervened to promote overweight and obese children's eating behavior. Intervention programs aimed at promoting healthy eating behaviors among children, thereby mitigating the risk of pediatric obesity, should primarily target parents. Additionally, these programs should specifically address children aged 9–12 or whose fathers are unemployed. Future research endeavors should further investigate the influence of these factors on eating behavior, exploring intricate associations and underlying mechanisms. This study offers a pioneering theoretical foundation for interventions targeting overweight and obese children's eating behaviors, thereby facilitating the promotion of healthy dietary habits. It is conducive to the promotion of healthy eating behavior, and has important significance for the prevention and treatment of childhood obesity.

## Data Availability

The raw data supporting the conclusions of this article will be made available by the authors, without undue reservation.
