# Supplementary material for: Individual, family and social-related factors of eating behavior among Chinese children with overweight or obesity from the perspective of family system
Source: Front Pediatr. 2024 Feb 22;12:1305770. doi: 10.3389/fped.2024.1305770 (PMC10917944; doi:10.3389/fped.2024.1305770)
Supplement: Supplementary file 1 [file Table1.pdf]

Supplementary Materials

Supplementary Table 1. Criteria for children aged 6–12 years to screen overweight and obesity

	Boys		Girls	
Age(years)	Overweight	Obesity	Overweight	Obesity
6.0～	16.4	17.7	16.2	17.5
6.5～	16.7	18.1	16.5	18
7.0～	17	18.7	16.8	18.5
7.5～	17.4	19.2	17.2	19
8.0～	17.8	19.7	17.6	19.4
8.5～	18.1	20.3	18.1	19.9
9.0～	18.5	20.8	18.5	20.4
9.5～	18.9	21.4	19	21
10.0～	19.2	21.9	19.5	21.5
10.5～	19.6	22.5	20	22.1
11.0～	19.9	23	20.5	22.7
11.5～	20.3	23.6	21.1	23.3
12.0～	20.7	24.1	21.5	23.9
